# Determinants and protective behaviours regarding tick bites among school children in the Netherlands: a cross-sectional study

**DOI:** 10.1186/1471-2458-13-1148

**Published:** 2013-12-09

**Authors:** Desiree JMA Beaujean, Fedor Gassner, Albert Wong, Jim E Steenbergen van, Rik Crutzen, Dirk Ruwaard

**Affiliations:** 1 National Institute for Public Health and the Environment, Centre for Infectious Disease Control, P.O. Box 1, 3720, BA Bilthoven, The Netherlands; 2 Department of Statistics, Mathematical Modeling and Data Logistics, National Institute for Public Health and the Environment, P.O. Box 1, 3720, BA Bilthoven, The Netherlands; 3 Centre for Infectious Diseases, Leiden University Medical Centre, P.O. Box 9600, 2300, RC Leiden, The Netherlands; 4 Department of Health Promotion, Maastricht University, CAPHRI School for Public Health and Primary Care, P.O. Box 616, 6200, MD Maastricht, The Netherlands; 5 Department of Health Services Research, Maastricht University, CAPHRI School for Public Health and Primary Care, P.O. Box 616, 6200, MD Maastricht, The Netherlands

**Keywords:** Perceptions, Lyme borreliosis, Lyme disease, Ticks, Tick bites, School children, Prevention, Protective behaviour, Knowledge

## Abstract

**Background:**

Lyme borreliosis (LB) is the most common tick-borne disease in the United States and Europe. The incidence is 13.4 per 100,000 inhabitants in the United States and more than 300 per 100,000 inhabitants in Europe. Children are at highest risk of LB. In the Netherlands in 2007, the incidence of tick bites in children between 10–14 years varied from 7,000 -11,000 per 100,000, depending on age. This study among Dutch school children aimed to examine the knowledge, perceived threat, and perceived importance of protective behaviour in relation to tick bites and their potential consequences.

**Methods:**

In April 2012, the municipal health services (MHS) contacted primary schools to recruit children 9–13 years by telephone, e-mail, or advertisement in MHS newsletters. In total, 1,447 children from 40 schools participated in this study by completing a specifically developed and pretested compact paper questionnaire. Regression models were used to determine which covariates (e.g. forest cover, previous education, knowledge) are associated with our response variables.

**Results:**

70% (n = 1,015) of the children answered at least six out of seven knowledge questions correctly. The vast majority (93%; n = 1345) regarded body checks as very or somewhat important, 18% (n = 260) was routinely checked by their parents. More frequent body checks were associated with good knowledge about ticks and tick-borne diseases and knowing persons who got ill after tick bite. Children in areas with a higher forest cover were more likely to be checked frequently.

**Conclusions:**

Most children have a good knowledge of ticks and the potential consequences of tick bites. Knowing persons who personally got ill after tick-bite is associated with a good knowledge score and leads to higher susceptibility and better appreciation of the need for body checks. Perceived severity is associated with a good knowledge score and with knowing persons who got ill after tick-bite. Is seems to be useful to additionally address children in health education regarding ticks and tick-borne diseases. The relationship between health education programs for children (and their parents) about ticks and their possible consequences and prevention of these deserves further study.

## Background

Lyme borreliosis (LB) is the most common tick-borne disease in the United States (USA) and Europe. From 1992 through 2006, a total of 248,074 cases of LB were reported to the U.S. Centers of Disease Control and Prevention, with a nationwide incidence of 13.4 per 100,000 inhabitants. The annual count increased 101%, from 9,908 cases in 1992 to 19,931 cases in 2006. Incidence was highest among children aged 5--14 years [1]. In Europe, where the main endemic areas are located in Scandinavia and the south central areas of Germany, Austria, north-east Italy, and Slovenia, the reported incidence is more than 300 cases per 100,000 inhabitants. In the Netherlands, the rate of general practitioner (GP) consultations for tick bites increased from 191 per 100,000 persons in 1994 to 564 per 100,000 persons in 2009 [2]. In 1994, patients visiting the GP for erythema migrans (EM), a circular red skin rash around the place of the tick bite, was estimated at 39 per 100,000 inhabitants. This rate increased to 134 per 100,000 in 2009 [2], and similar rises occurred in other European countries as well [3].

Children are at highest risk of LB, with a peak incidence rate among boys aged 5–9 years [4,5]. In the Netherlands, a repeated retrospective study among general practitioners has shown a continuing and strong increase in consultations for tick bites and for EM between 1994 and 2009 [2,6,7]. The increasing numbers of tick bites, adding up to 1.5 million people with a tick bite in 2009 [1], poses a progressive threat to public health. As these data was derived from general practices, the incidence of tick bites is probably higher in the wider population, which includes people not routinely visiting a GP. The Dutch data accords with findings of an LB seroprevalence survey conducted in children throughout Germany [8] that point to children as a distinct and vulnerable risk group [9]. Another study found that children aged 5–14 years are at higher risk for LB in Europe. As in the Netherlands, EM is the most reported manifestation of LB (77-89%) in children across Europe [10].

This complex infection has a number of objective manifestations, including a characteristic skin lesion called erythema migrans (the most common presentation of early Lyme disease), certain neurologic and cardiac manifestations, and pauciarticular arthritis (the most common presentation of late Lyme disease), all of which usually respond well to conventional antibiotic therapy. Despite resolution of the objective manifestations of infection after antibiotic treatment, a minority of patients have fatigue, musculoskeletal pain, difficulties with concentration or short-term memory, or all of these symptoms [11].

In addition, there is an extensive range of rare manifestations [12-16]. The increase of LB might be caused by changes in pathogen and vector populations but could also reflect increased awareness. Indeed, the more citizens and medical personnel are aware of LB, the more LB is diagnosed [17]. However, LB can be significantly more difficult to identify in children, because half of all EM is situated in head and neck and can go unnoticed and late manifestations can present with non-specific chronic complaints [14].

Health education is considered the most important approach for preventing LB, because no vaccine is available and effective measures for controlling tick populations are experimental or insufficient [18]. In the Netherlands, health education materials focus on personal behaviours [19]: avoiding tick-infested areas, wearing protective clothing (e.g. long-sleeved shirts and long trouser pants to minimise exposed skin), using tick repellents on skin or clothing, doing body checks after being outdoors. Except for body checks, these measures are not well accepted by the general public in the Netherlands [20,21].

At present, health education materials and LB prevention research are mainly aimed at adults. Although parents are the designated persons to check children for ticks, the need for checking should be communicated directly to children as well [20]. Teaching them to recognise the tiny nymphal stage of ticks and the features of tick habitats can encourage them to urge parents to do timely body checks [18]. Therefore it seems logical to develop education materials aimed at both children and their parents. To develop tailored education materials it is necessary to determine the determinants associated with the risk behaviours. This study among Dutch school children aimed to examine the response variables of knowledge, perceived threat, and perceived importance of protective behavior in relation to tick bites and their possible consequences. The study is based on the Protection Motivation theory [22] This theory posits that a ‘threat appraisal’ is formed by an individual based on the perceived likelihood of a particular event (denominated here as ‘perceived susceptibility’) and its perceived severity. The way in which individuals choose to respond to a threatening situation is termed their ‘coping appraisal’. It is based on their belief that a recommended behaviour will resolve the threat ‘response efficacy’) and their belief that they are able to perform the behaviour (‘self-efficacy’). The latter belief is less important in the current study, because parents must perform the behaviour (e.g. tick checks). Furthermore, since our subjects are primary school children, the questions must be limited in number and easily understood by children.

## Methods

### Participants

This study was performed in April 2012 among Dutch school children aged 9–13 years, attending the two highest grades of primary school. To request voluntary participation, municipal health service (MHS) workers contacted schools in their region by telephone, e-mail, or advertisement in MHS newsletters. The schools could participate voluntarily. School children were recruited at school-level; all children in the targeted age-group in these schools participated in the study (except in case of illness or absence). Approximately 1,100 schools were reached, resulting in a convenience sample of 40 schools nationwide. The study included 1,447 children. Being a general survey among healthy volunteers from the general population, it did not require formal medical ethical approval, according to Dutch law [23].

### Questionnaire

A concise two-sided paper questionnaire with 12 questions was developed and pretested to make it accessible for primary school children of varying education levels (see questions in Additional file 1). Answer categories were minimised to a three-point scale; text was limited to short sentences, and images were used when possible. We included the following constructs: knowledge (assessed by asking 7 questions on tick ecology, basic prevention, and tick bites); perceived severity (asking about the possible consequences of a tick bite); perceived susceptibility (asking whether the child thinks he/she could personally become ill after a tick bite); an additional proxy for perceived susceptibility (asking whether the child personally knows someone who became ill after a tick bite); perceived importance of protective behaviour as a proxy for response efficacy (asking whether the child thinks tick-checks are important), and actual protective behaviour (asking for the frequency of tick checks performed by his/her parents). Finally children were asked if they had had previous classroom lectures on ticks. Teachers handed out the questionnaires, which were completed in the classroom, and sent them back by regular mail*.*

### Analysis

For each construct we used a set of covariates which were selected based on proven or plausible effects of covariates on an outcome category, as described below and in Table 1. Most covariates were obtained through the questionnaire, but two additional covariates were collected as a measure of tick habitat exposure: the level of urbanisation and the level of forestation.

**Table 1:** Overview of model characteristics per domain

**Response variate**	**Outcome variable *Y***	**Outcome categories *j***	**Model type**	**Covariates**
Knowledge	Knowledge sufficiency (based on Q2-8)	*j = 1 (* “Sufficient”) if at least 6 out of 7 questions have been answered correctly; *j = 0* “Insufficient”)	Logistic regression with random effects	Forest cover (%), Urbanisation level (scale 1–5); Previous education; Perceived susceptibility (knowing persons with LB).
Perceived severity	Consequence of tick bite: disease or itch (based on Q5)	*j = 1* (“Disease” ),	Logistic regression with random effects	Forest cover (%), Urbanisation level (scale 1–5); Previous education; Knowledge score ≥5; Perceived susceptibility (knowing persons with LB).
		*j = 0* (“Itch” or “Not sure”)		
Perceived susceptibility	Can you get ill? (based on Q9)	*j = 2* (“Yes”),	Ordered logit with random effects	Forest cover (%), Urbanisation level (scale 1–5); Previous education; Knowledge score ≥6; perceived severity; Perceived susceptibility (knowing persons with LB); Protective behaviour.
		*j = 1* (“Not sure”),		
		*j = 0* (“No”)		
Perceived importance	Body checks important? (based on Q10)	*j = 2* (“Very important”),	Ordered logit with random effects	Forest cover (%), Urbanisation level (scale 1–5); Previous education; Knowledge score ≥6; Perceived severity; Perceived susceptibility (Can you get ill and Knowing persons with LB).
		*j = 1* (“Somewhat important”),		
		*j = 0* (“Not important”)		
Protective behaviour	Body check frequency (based on Q12)	*j = 2* (“Often”),	Ordered logit with random effects	Forest cover (%), Urbanisation level (scale 1–5); Previous education; Knowledge score ≥6; Perceived severity; Perceived susceptibility (Can you get ill and Knowing persons with LB);Perceived importance.
		*j = 1* (“Sometimes”),		
		*j = 0* (“Never”)		

All *j = 0* are considered reference categories in each model.

In the Unites States, knowledge and behaviour on LB prevention has been associated with the level of urbanisation [24]. Therefore, data on the extent of urbanisation based on a 1–5 ordinal scale of household density per postal code area was extracted from the Dutch National Atlas of Public Health [25]. Here, level 1 represents a highly urbanised area and level 5 represents the lowest population density (i.e. rural). The public perception of tick-risk areas is mostly related to woodlands [26], as is LB risk [27]. Therefore, a variable to estimate the children’s perception of tick exposure was constructed by assessing the percentage of forest cover (deciduous and evergreen) in a radius of 10 km around each studied school, according to the Dutch land use database 2007–2008 [28]. These distances reflect the fact that children live close to their primary school, which on average is 0.6 km in the Netherlands [29].

**Figure 1:**
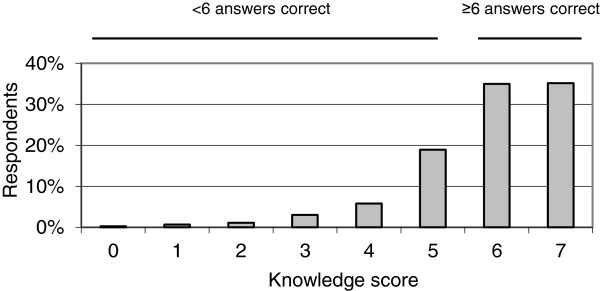
**Knowledge level in Dutch primary school children aged 9–13 years (N = 1447).** The “knowledge score” on the x-axis represents the number of correct answers out of seven knowledge questions.

The class of generalised linear mixed models (GLMM) was used to explore the relationship between the constructs assessed in the questionnaire and the sets of covariates, whilst accounting for the cluster structure in our data [30]. Children attending the same school are likely to score similarly on the constructs, and can therefore be considered as a cluster. GLMMs adjust for this correlation within clusters by introducing a random effect; more specifically, we used a random intercept for schools. We observed a clear subdivision where a group of respondents answered 6 or 7 out of 7 knowledge questions correctly, or answered 5 or fewer questions correctly (Figure 1). The knowledge construct was therefore operationalised as a binary variable, with 6–7 of 7 questions correct was considered Sufficient (i.e. good) and 5 or fewer as Insufficient. For this construct, we used a GLMM assuming a Bernoulli distribution and logit link (i.e. logistic regression with random effects). Note that we could have also operationalised the construct as Sufficient, Somewhat sufficient, and Insufficient; but as shown in the results section, most children answered most questions right, making a such a detailed categorisation less appropriate.

The other constructs were operationalised by ordered outcome variables with three outcome categories; for these constructs, we considered ordered logit/cumulative logit models with random effects [31], in which the probability of each category is assumed to be dependent on a set of covariates. Note that we assume ‘No’, ‘Don’t know’ and ‘Yes’ to be ordered, with ‘Don’t know’ falling between ‘No’ and ‘Yes’, as it indicates doubt between them^a^. These models assume ‘proportional odds’, which implies that only one set of regression coefficients, is applicable for all outcome categories (with the exception of the intercept). Using the Likelihood Ratio test, we found that this assumption was not violated, except for the protective behaviour construct. For this construct we considered a GLMM with multinomial distribution, but found that it led to convergence problems, presumably due to model complexity. We therefore retained the ordered logit model for this construct in order to adjust for the cluster structure. The specific model characteristics (model type, distribution, covariates, outcome categories) used per construct are summarised in Table 1.

A difficulty arises in the interpretation of the effect sizes for each covariate, as our GLMM models are non-linear. To facilitate interpretation, we make multiple predictions with our estimated models. For instance, if we are interested in the effect size of one covariate, we first make a prediction of the probability of a given response category for a baseline value of this covariate while assuming all other covariates assume their mean or modus values. Then we change the value of the covariate and make a new prediction while assuming all other covariates remain constant (ceteris paribus). The change in the predictions can be interpreted as the difference in risk that is associated with a change in the covariate, indicating the effect size under the ceteris paribus assumption. In the remainder of this article we will therefore refer to this risk difference as the ‘effect size’. The absolute value of an effect size can vary between zero and one. Whether or not an effect is considered large depends strongly on context, as will be illustrated in the next section. The statistical software R [32] was used for our analyses.

## Results

The study included 1,447 children from 40 primary schools, with an average of 36 (range: 10 to 106) participating from each school. The schools were geographically dispersed across the country, there being 14 with 420 children in the Northern part of the country, 21 with 814 children in the Middle, and 5 with 213 children in the Southern part. In general, response rates to the questions were high, with 96.7% (n = 1,403) of children completing the questionnaire without skipping any questions; 2.9% (n = 42) skipping one question, and 0.14% (n = 2) skipping 5 out of 12 questions (Table 1). Data of all respondents were included in the dataset; missing data (e.g. where a participant skipped a question) were not imputed.

### Knowledge and perceived severity

**Figure 2:**
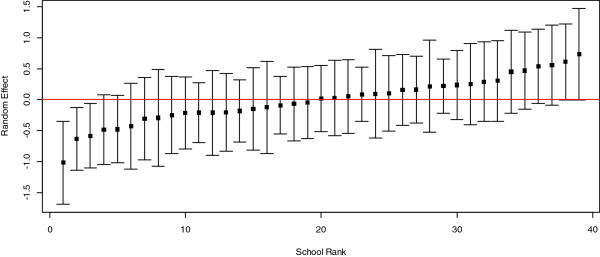
**Variation in knowledge level by school, illustrated by the posterior empirical bayes estimates of the random effects for each school rank (squares), with their 95%confidence intervals.** Red horizontal line indicates the level for the average school; if an interval does not intersect with the red line, the corresponding school differs significantly from the average.

Of the respondents, 78% (n = 1,131) indicated that they never had classroom education about ticks (Table 2). The vast majority, 70% (n = 1,015) nevertheless had a sufficient knowledge, as they answered at least six out of seven knowledge questions correctly (Figure 1). The favorite habitat of ticks, whether one can become ill after a tick bite (also interpreted as perceived severity), and the shape of unfed ticks were the three questions most often answered correctly (98%, n = 1,419; 94%, n = 1,353 and 87%, n = 1,255, respectively). Our logistic regression model with random effects indicates that the variation in knowledge among schools was considerable (see Figure 2). Prior tick-related education is significantly associated with a higher probability of answering at least six questions correctly (p < 0.01). As the corresponding estimated effect size is 0.096, the probability of answering at least six questions correctly is 0.096 higher when having prior education, assuming all other covariates remain at their mean or modus level. Also, knowing persons with LB, here used as proxy for perceived susceptibility, was significantly associated with a higher knowledge level (p < 0.001, effect size 0.117), but forest cover and level of urbanisation were not (Table 3). Perceived severity, i.e. being aware of possible consequences of tick bites, was associated with a good knowledge score (answering ≥5 questions correctly, p < 0.001, effect size 0.076) and with knowing persons with LB (p = 0.003, effect size 0.087).

**Table 2:** Questionnaire responses of 1,447 respondents

**Question (determinant)**	**Abbreviated question**	Correct answer/yes/*	Incorrect answer/no/*	Don’t know/*	**No response (n)**
1	Previously educated on ticks at school	21,4%	78,2%	n.a.	0,4% (6)
2 (K)	How to recognise an unfed tick (Image: tick shape or ant shape)	86,7%	6,6%	6,4%	0,2% (3)
3 (K)	Unfed tick size estimation (Image: 1 mm dot or 10 mm dot)	82,2%	9,3%	7,5%	1,0% (14)
4 (K)	Tick habitat (Image: forest or paved playground)	98,1%	0,7%	0,7%	0,6% (8)
5 (K;PV)	Consequence of tick bite (disease or itch)	93,5%	4,1%	2,4%	0,1% (1)
6 (K)	Where ticks reside (near ground or up in trees)	64,2%	29,1%	6,6%	0,1% (1)
7 (K)	How to prevent tick bite (tick check or washing)	86,1%	8,5%	5,3%	0,1% (2)
8 (K)	Main bite sites on the body (image: hairline, armpits, groin and knees; or mouth, chest, fingers and toes)	75,2%	16,9%	7,7%	0,2% (3)
9 (PS)	Perceived susceptibility for LB (can you personally get ill after tick-bite?)	68,6%	16,7%	14,4%	0,3% (4)
10 (PI)	Importance of tick check (not, somewhat, or very important)	*7,0%	*52,5%	*40,4%	0,2% (3)
11 (PSK)	Perceived susceptibility (knowing persons with illness after tick bite)	26,7%	66,6%	6,4%	0,3% (4)
12 (PB)	Checked for tick bites to date? (never, occasionally, every time)	*30,1%	*51,8%	*17,9%	0,2% (3)

Behavioural determinants are given where relevant by abbreviations: *K* knowledge; *PV* perceived severity; *PS* perceived susceptibility; *PSK* perceived susceptibility due to knowing persons with LB; *PI* perceived importance, and *PB* protective behaviour.

*See abbreviated question for answer category.

**Table 3:** Model coefficients β and p-value (p) for each determinant and response variable

**Covariate**	**Knowledge score**	**Perceived severity (q5)**	**Perceived susceptibility**	**Perceived importance**	**Protective behaviour**
% Forest cover	2.50 (0.20)	0,15 (0.96)	0.68 (0.60)	-2.63 (*)	4,54 (**)
Urbanisation level 2	0.11 (0.82)	-0,34 (0.62)	-0,61 (0,08)	-0.39 (0.26)	-0.33 (0.34)
Urbanisation level 3	0.12 (0.82)	-0.12 (0.88)	-0.25 (0.6)	-0.05 (0.87)	0.40 (0.24)
Urbanisation level 4	0.12 (0.82)	0,49 (0.60)	-0.39 (0.28)	-0.98 (*)	-0.32 (0.38)
Urbanisation level 5	0.12 (0.78)	-0,30 (0.67)	-0.60 (0.06)	-0.53 (0.09)	0.36 (0.25)
Previous education	0.45 (*)	-0.04 (0.90)	0.02 (0.91)	0.13 (0.34)	0.13 (0.35)
Knowledge score ≥6	nd	1.78 (**)†	0.13 (0.37)	0.42 (**)	0.61 (**)
Perceived severity (is aware of tick bite consequence)	nd	nd	(0.69) (*)	-0.08 (0.74)	0.31 (0.21)
Perceived susceptibility (doesn’t know)	nd	nd	nd	0.28 (0.16)	-0.45 (*)
Perceived susceptibility (can get ill)	nd	nd	nd	0.55 (**)	-0.08 (0.58)
Perceived importance (a bit important)	nd	nd	nd	nd	0.99 (**)
Perceived importance (very important)	nd	nd	nd	nd	2.16 (**)
Perceived susceptibility (not knowing person with LB)	-0.23 (0.35)	-0.67 (0.07)	-0.04 (0.85)	-0.36 (0.12)	-0.39 (0.10)
Perceived susceptibility (knowing person with LB)	0.57 (**)	1.17 (*)	0.58 (**)	0.45 (**)	0.37 (*)
Protective behaviour (sometimes)	nd	nd	0.17 (0.21)	nd	nd
Protective behaviour (frequently)	nd	nd	0.29 (0.13)	nd	nd

An *indicates p ≤ 0.05, and **indicates p ≤ 0.001. Those variables that were not included in analyses per domain are indicated with “nd”.

† This model was run with an adjusted knowledge score, where question 5 was excluded and the criterion was set at ≥5 questions correct.

### Perceived susceptibility

Of the respondents, 69% (n = 992) believed they were susceptible to illness due to a tick bite. Children aware of possible consequences of a tick bite (perceived severity) are more likely to think that they could personally get ill after a tick bite (p < 0.001). Given the effect size of 0.116, the probability of perceiving oneself as susceptible increases by 0.116 when being aware of the possible consequences. In line with knowledge score, the proxy for perceived susceptibility (knowing persons with LB) was positively associated with the perceived susceptibility of personally becoming ill after a tick bite (p < 0.001, effect size 0.117). A quarter of the respondents (27%, n = 386), indicated knowing persons with a past or present episode of LB, a secondary measure of perceived susceptibility (Table 2). However, forest cover, level of urbanisation, and the frequency of body checks were not associated with perceived susceptibility (Table 3).

### Perceived importance of protective behaviour and actual practice of protective behaviour

The vast majority (92% n = 1,343) of the respondents regarded body checks as very or somewhat important (40% and 52%, respectively). This finding was associated with a good knowledge level (p = 0.001, effect size 0.098, which suggests that the probability of finding body checks very important increases by 0.098 with a good knowledge level). Additionally, a high perceived susceptibility (p < 0.001, effect size 0.124) and a high proxy for perceived susceptibility through knowing persons with LB (p < 0.001, effect size 0.112) were associated with finding body checks very or somewhat important. Surprisingly, respondents from areas with a high forest cover within a 10 km radius around the school were less likely to find tick checks important (p = 0.049, with a large effect size of -0.392) than children from less forested areas*.* In line with this observation, but with smaller effect size: the less urbanised area level 4 also shows this effect (significant at p = 0.02, effect size -0.191). The least urbanized level 5 was not significant (p = 0.09, effect size -0.134).

Half of the children (52%, n = 749) reported being inspected occasionally for tick bites after having been outside. Nearly one third of the total respondents (30%, n = 436) had never had a body check, and only 18% of the respondents indicated they were checked after every visit to nature. Children with a good knowledge score (p < 0.001, effect size 0.053) and those knowing persons with LB (proxy for perceived susceptibility, p = 0.004, effect size 0.046) were more likely to be checked often. Likewise, those who considered body checks somewhat or very important were checked more frequently (both p < 0.001, effect size 0.075 and 0.429, respectively). Respondents in areas with a higher forest cover were more likely to be often checked for ticks (p < 0,001, effect size 0.810), which implies that their parents perceived susceptibility even if the children did not.

## Discussion

We studied school children’s knowledge, perceived severity, perceived susceptibility, and protective behaviour in relation to ticks and their possible consequences in the Netherlands, where LB incidence has increased sharply throughout the past two decades. We conclude that most Dutch children have very good general knowledge about ticks and their possible consequences, although three-quarters of them never had classroom lectures about ticks. Seventy percent answered at least six out of seven knowledge questions correctly. This percentage is higher than among adults studied in tick- and LB-endemic areas [21,33] and may reflect discussion of ticks and their possible consequences by children’s television programs in recent years.

An exception to the good general knowledge was that most children incorrectly believe that ticks live in trees and that headgear therefore offers some protection. This common misunderstanding exists also among adults [21] and must be addressed in education programs for all age groups. Awareness that ticks occur in lower vegetation is essential for understanding why protective clothing includes long trousers and shoes with socks, not caps. Such awareness also enables more effective body checks.

Knowing persons who personally got ill after tick bite was significantly associated with a higher knowledge level. Knowing such persons was found by others to predict specific tick-bite protective behaviour [34]. Perceived severity (based on q5: being aware of tick bite consequences) was likewise associated with higher knowledge scores. Sixty-nine percent of the children perceived themselves as susceptible for illness due to a tick bite. They were aware of the possible consequences of a tick bite, and such awareness is crucial to a realistic perception of personal risk. People with a realistic perception of risk are more likely to be motivated to engage in preventive behaviour [34,35].

The evidence base for checking for ticks to prevent LB is still limited, but there is some evidence. Smith and Jacobs showed in their studies that checking the skin for ticks during outdoor activities and removing them within 24 hours, reduced the chance of getting Lyme disease (odds ratio 0.59; 90% CI 0.48-0.72, respectively 0.6%, 95% CI: 0.0003-0.029) [36,37]. The vast majority (93%) of children regarded body checks as somewhat or very important. Children with a sufficient knowledge level, a high perceived susceptibility, or knowing persons who got ill after tick bite were more likely to find body checks very or somewhat important and were also checked by their parents more frequently. Whereas nearly 18% of the children indicated being inspected after every nature visit, the rest (82%) were checked occasionally (n = 749, 52%) or never (n = 436, 30%). In comparison, 28% of the Brazilian population on the Massachusetts idland of Martha’s Vineyard checks their skins for ticks routinely [33]*.* Dutch children in the least urbanised and most forested areas regarded tick checks as less important, but were nevertheless checked for tick bites more frequently than more urban children. Clearly the less urbanised parents are more vigilant, but their children may be so accustomed to having the checks (habitual behavior) that they give them less notice than do urban children, for whom they are less routine*.*

Our findings suggest that it is useful to focus on children in health education regarding ticks and tick-borne diseases. Although the parents must perform body checks on children, the knowledge, perceived susceptibility, and perceived importance of protective behaviours among children is related to the desired behaviour of the parents: performing body checks.

This is the first study to evaluate the knowledge, perceived severity and susceptibility, and protective behaviour of school children in relation to ticks and their possible consequences. As to possible limitations, selection bias may have occurred because primary schools in the southern Netherlands had holiday during the study period. However, with no reason to believe that those schools differ in tick-related knowledge from the schools that did participate, we think this will not affect the generalisability of the results.

It could be a limitation that our questionnaire-based approach kept the number of questions to 12. Additional questions on the studied constructs and on the protective measures taken by the respondents to prevent tick bites may have been beneficial in terms of validity, but was deemed less feasible for primary school children. Since this is the first study among school children we developed and pretested a new questionnaire. This questionnaire needs to be validated in future studies (e.g., in terms of the accuracy of the reported behaviors).

The question: “Do you know someone who has become ill after a tick bite?” might be interpreted as if a tick bite preceding development of Lyme disease will always be recognized. However, the majority of patients who develop Lyme disease do not report a preceding tick bite. Therefore, this question might be ambiguous. Since we used this question too as a proxy for the perceived severity of the children, we think this ambiguity is acceptable.

Finally, this cross-sectional study could not demonstrate a cause-effect relationship between the determinants in the children and the behaviour of the parents. For example, it may be that body checks by parents lead to higher knowledge, perceived severity and susceptibility among children, but equally it may be that these factors in children lead to parents’ performing body checks. Ideally we might have included both the school children and their parents, performing repeated measurements to analyse differences in the knowledge, perceived threat, and behaviours regarding ticks and their possible consequences within the family, showing a more plausible cause-effect relationship. Since the children in this study were contacted through their schools and not through families, it was complicated to include their parents.

## Conclusion

The previous studies on knowledge, perceived severity and susceptibility, and protective behaviour regarding ticks and their possible consequences have focussed on adults or the general population. As the first to focus solely on primary school children, our study can assist in development of education programs on ticks for children, a high risk group in Western Europe and the USA*.* Such programs should reflect our findings that good knowledge of ticks and knowing someone who got ill after tick bite lead to more body checks. Programs should take into account that children in forested areas view checks as less important, but are checked more frequently by parents. Further research on the relationship between health education programs about ticks aimed at children (and their parents) in order to prevent LB is needed.

## Endnote

^a^If we do not make this assumption, and fit a logistic regression model with random effects (with only “no” and “yes” as outcome categories, and “don’t know” omitted), we find that the results are very similar.

## Competing interests

The authors declare that they have no competing interests.

## Authors’ contributions

All authors contributed to the study design. DB and FG played major roles in the data collection process. Data analysis was performed by AW, FG and DB. DB, FG and AW wrote the first draft of the manuscript. JvS, RC and DR critiqued the manuscript and contributed to further drafts. All authors read and approved the final manuscript.

## Pre-publication history

The pre-publication history for this paper can be accessed here:

http://www.biomedcentral.com/1471-2458/13/1148/prepub

## Supplementary Material

Additional file 1 Questionnaire. Click here for file
