# Supplementary material for: Determinants and protective behaviours regarding tick bites among school children in the Netherlands: a cross-sectional study
Source: BMC Public Health. 2013 Dec 9;13:1148. doi: 10.1186/1471-2458-13-1148 (PMC3907142; doi:10.1186/1471-2458-13-1148)
Supplement: Additional file 1 — Questionnaire. [file 1471-2458-13-1148-S1.docx]

**Additional file 1**

Questionnaire

What age are you?

Multiple choice questions (Instruction to tick one box in each question):

1. Did you ever receive classroom lectures about ticks?
   1. Yes
   2. No
2. What does a tick look like?
   1. Image: Silhouette of an ant
   2. Image: Silhouette a tick
   3. I don’t know
3. What is the real size of a tick that could bite you?
   1. Image: poppy-seed size black dot
   2. Image: pea size black dot
   3. I don’t know
4. At what sort of place would you expect to encounter ticks?
   1. Color image of path in a forest
   2. Color image of sand-covered playground
   3. I don’t know
5. Why should you watch out for ticks?
   1. Ticks make you itch
   2. Ticks can make you ill
   3. I don’t know
6. At what place do ticks prefer to live?
   1. Up in the trees
   2. Near the ground in brushes and tall grass
   3. I don’t know
7. What can you do to prevent tick bites?
   1. Wash carefully to rinse off ticks
   2. Check your clothes and body with an adult and pull out attached ticks
   3. I don’t know
8. Which picture shows best where ticks prefer to bite?
   1. Image: child body contours with arrows pointing at nose, chest, hands and toes
   2. Image: child body contours with a rows pointing at ears, armpits crotch and knees
   3. I don’t know
9. Do you think that you could personally become ill after a tick bite?
   1. Yes
   2. No
   3. I don’t know
10. Do you consider it important to be checked for tick bites after playing in an area where ticks may live?
    1. Not important
    2. Somewhat important
    3. Very important

11) Do you know someone who has become ill after a tick bite?

- 1. Yes
  2. No
  3. I don’t know

12) Did you ever do a body inspection for tick bites with your parent or carer?

- 1. Never
  2. Occasionally
  3. Every time after a visit to an area where ticks may live
